# The Association of Sleep Hygiene and Drowsiness with Adverse Driving Events in Emergency Medicine Residents

**DOI:** 10.5811/westjem.2020.8.47357

**Published:** 2020-10-27

**Authors:** Walter Green, Xiang Gao, Kaigang Li, Barbara C. Banz, Jia Wu, Michael J. Crowley, Deepa R. Camenga, Federico E. Vaca

**Affiliations:** # Yale Developmental Neurocognitive Driving Simulation Research Center (DrivSim Lab); * Yale School of Medicine, Department of Emergency Medicine, New Haven, Connecticut; † Colorado State University, Department of Health and Exercise Science, Fort Collins, Colorado; ‡ Yale Child Study Center, New Haven, Connecticut

## Abstract

**Introduction:**

Prior research shows that physicians in training are at risk for drowsy driving following their clinical duties, which may put them in danger of experiencing adverse driving events. This study explores the relationship between sleepiness, overall sleep hygiene, level of training, and adverse driving events following an overnight shift in emergency medicine (EM) residents.

**Methods:**

Throughout the 2018–2019 academic year, 50 EM residents from postgraduate years 1–4 completed self-administered surveys regarding their sleepiness before and after their drive home following an overnight shift, any adverse driving events that occurred during their drive home, and their overall sleep hygiene.

**Results:**

Fifty out of a possible 57 residents completed the survey for a response rate of 87.7%. Sleepiness was significantly associated with adverse driving events (beta = 0.31; P < .001). Residents with high sleepiness levels reported significantly more adverse driving events. Residents reported significantly higher sleepiness levels after completing their drive home (mean = 7.04, standard deviation [SD] = 1.41) compared to sleepiness levels before driving home (mean = 5.58, SD = 1.81). Residency training level was significantly associated with adverse driving events (beta = −0.59, P < .01). Senior residents reported significantly fewer adverse driving events compared to junior residents.

**Conclusion:**

Emergency physicians in training are at risk for drowsy driving-related motor vehicle crashes following overnight work shifts. Trainees of all levels underestimated their true degree of sleepiness prior to initiating their drive home, while junior residents were at higher risk for adverse driving events.

## INTRODUCTION

The dangers of sleep deprivation are featured by the negative effects on multiple facets of neurocognitive function, including higher level executive function and working memory.^1^ One of the most severe consequences of sleep deprivation is drowsy driving that may result in property damage, injury, and fatal crashes. Between 2011–2015, drowsy driving was implicated in 4121 fatalities related to motor vehicle-related crashes.^2^ Drowsy driving is well known to compromise not only decision-making while driving, but also the driver’s ability to control the vehicle.^3^ In simulated driving studies, drivers who are sleep deprived show poorer performance on driving tasks compared to individuals who consumed a moderate amount of alcohol.^3^–^5^ However, relatively little media attention and research are focused on advancing this special area of impaired driving (ie, compared to alcohol or drug-impaired driving) that has important implications for the public’s health.

The trend of motor vehicle crashes (MVC) due to daytime drowsiness continues to rise and has led to an increased interest in sleep hygiene – an assessment of an individual’s sleeping habits that influence his or her sleep experience.^6^ Research shows a strong positive relationship between an individual’s sleep hygiene and the quality of his or her sleep.^6^,^7^ That is, individuals with worse sleep hygiene are expected to experience poorer sleep quality (ie, less restful sleep). Certain individuals with poor sleep hygiene, such as night shift workers, are more likely to develop and accumulate sleep deprivation, in turn putting them in danger of experiencing adverse events on the job or outside of the workplace, including drowsy driving-related crashes.

Medical professionals have a particularly increased risk of sleep deprivation and poor sleep hygiene due to the rigor of their work (eg, physical, emotional, cognitive) and systematic requirements to work extended hours and overnight shifts. Young physicians in training are especially vulnerable to drowsy driving and subsequent MVCs as they often work shifts that last longer than 24 hours or frequently transition between day and overnight work shifts.^8^–^10^ Recent regulations that limit resident physician duty hours have focused on improving patient safety,^11^ but these regulations also resulted in a greater need to transition frequently from day to overnight work shifts, a pattern that may further detrimental and worsen sleep deprivation.

Among medical trainees, emergency medicine (EM) residents may be at higher risk for both poor sleep hygiene and drowsy driving with subsequent adverse driving events (eg, drifting out of the roadway lane; unexpectedly braking hard to avoid rear-ending a vehicle; running a stop sign). Due to the nature and contextual setting of their work, they regularly have a high number of sporadic overnight shifts, leading to more opportunities for cumulative sleep deprivation and drowsy driving. In fact, one study found that 80% of near-crashes and nearly 75% of MVCs involving emergency physicians in training occurred while driving home after an overnight shift.^12^ Higher levels of self-reported drowsiness prior to driving home have been shown to be associated with subsequent adverse driving events.^8^

In a broader context, a foundational principle of any EM training program relies on the concept of trainees becoming more competent and proficient, clinically and procedurally, as they advance in their training and gain experience year by year. This raises the question as to whether in the setting of medical training EM residents adapt their sleep hygiene year to year, so that they experience less drowsiness after their shift work and, more importantly, experience fewer drowsy-driving adverse events. In other words, “Is there adaptation that occurs over years of training that may be protective to the more senior EM residents compared to junior residents?”

**Population Health Research Capsule**

What do we already know about this issue?

*Drowsy driving is a leading cause of injury and fatal crashes. Long work hours and overnight shifts put physicians in training at risk for drowsy driving-related crashes*.

What was the research question?

What is the relationship between sleepiness, sleep hygiene, training level, and adverse driving events after overnight shifts?

What was the major finding of the study?

*Higher sleepiness levels were reported after driving home. Resident training level was associated with adverse driving events*.

How does this improve population health?

*Our findings have implications for the well-being of emergency physicians in training as well as injury prevention programs focused on their safety*.

While the question may be intriguing, there remains a paucity of research assessing this important and highly relevant physician safety concern. It is possible that work experience could ameliorate overall sleep hygiene or, to some extent, the degree of drowsiness experienced after a night shift. Furthermore, it is unclear how EM residents manage sleep hygiene in general, and whether sleep hygiene is correlated with the degree of drowsiness experienced after a night shift. To fill this knowledge gap we aimed to examine the association between subjective sleepiness, level of training, overall sleep hygiene, and adverse driving events in EM residents after completion of an overnight shift.

## METHODS

### Recruitment and Design

We conducted a cross-sectional, self-administered online survey study. EM residents ranging in training levels from postgraduate years (PGY) 1–4 at a single, large, urban-based EM residency participated in the study throughout the 2018–2019 academic year. This study was approved by the university’s institutional review research board. All participants provided written informed consent.

Residents at this training program spend three months of the academic year working at a community hospital site 20 miles away from the main hospital. All residents have variability in working overnight shifts at this site at the discretion of the training program faculty scheduler. All overnight shifts are scheduled from 10 pm-7am requiring residents to travel approximately 20 miles home following their overnight shifts. Residents were sent a scheduled email the morning after an overnight shift at this community hospital with instructions to complete the online questionnaire after their drive home.

### Participants

A total of 50 EM residents completed the survey. Of these, three residents obtained a ride home and did not provide information regarding adverse driving events.

### Questionnaires and Measures

We used web-based survey software (Qualtrics XM, Provo, UT) to conduct the survey.^13^ Within the questionnaire, participants were asked to complete the Karolinska Sleepiness Scale,^14^ a validated measure of sleepiness. They rated their subjective level of sleepiness both before and after their drive home. The summed sleepiness scores (ie, before and after the drive home) were used for the linear regression analyses. Residents also completed the Sleep Hygiene Index,^6^ a validated instrument used to measure one’s stable sleep hygiene behaviors. Additionally, based on the investigator-developed Adverse Driving Events Questionnaire, participants were asked to evaluate their drive home after the overnight shift by answering “yes” or “no” to 15 questions that defined and quantified adverse/dangerous driving events. (See Appendix for all questionnaires used.)

### Statistical Analysis

We conducted a descriptive analysis of self-reported adverse driving events with baseline characteristics of EM residents. Thereafter, we performed a bivariate linear regression analysis to evaluate the association between subjective sleepiness and adverse driving events. These analyses were also used to assess the association of sleep hygiene with adverse driving events and sleepiness. A paired t-test was performed to compare the average level of sleepiness before and after driving home following an overnight shift. Finally, we conducted adjusted linear regressions to evaluate the relationship between sleepiness and adverse driving events while controlling for levels of residency training. Standardized Cronbach’s alpha of the Adverse Driving Events Questionnaire was calculated. All analyses were performed using SAS 9.4 (SAS Institute, Cary, NC).^15^ All levels of significance for the two-tailed tests of this analysis were set a priori as 0.05.

## RESULTS

Fifty out of a possible 57 residents completed the survey for a response rate of 87.7%. Table 1 shows the proportions and distribution of self-reported adverse driving events with baseline demographic characteristics of the EM residents. The PGY 1 + PGY 2 training levels were combined to reflect overall junior vs senior training levels (ie, PGY 3 and PGY 4). Among junior residents, 88% reported adverse driving events and the average number of reported driving events was 2.79 (SD = 1.89). Of the 14 residents at PGY 3 level, 79% reported adverse driving events and the average number of reported driving events was 2.57 (SD = 1.83). Of the 12 residents at PGY 4 level, 75% reported adverse events and the average number of reported driving events was 1.33 (SD = 0.98). Overall, the summed sleepiness score among the residents who reported adverse driving events was 13.37 (SD = 2.35). The standardized Cronbach’s alpha for the Adverse Driving Events Questionnaire was 0.97, suggesting that the items of this questionnaire have high internal consistency.

Table 2 shows the bivariate linear regression results. Sleepiness was significantly associated with adverse driving events (beta = 0.31; *P* < .001). Neither subjective sleepiness nor reporting of adverse driving events was found to be significantly related to sleep hygiene scores.

Table 3 shows the average level of sleepiness before and after driving home following an overnight shift. Residents reported significantly higher sleepiness levels after completing their drive home (mean = 7.04, standard deviation [SD] = 1.41) compared to sleepiness levels before driving home (mean = 5.58, SD =1.81).

Table 4 shows the results of the adjusted linear regression of adverse driving events on sleepiness, controlling for residency training levels. Both subjective sleepiness (beta = 0.30, *P* < .001) and residency training levels (beta = −0.59, *P* < .01) were significantly associated with adverse driving events. Residents with high sleepiness levels reported significantly more adverse driving events. Senior residents reported significantly fewer adverse driving events compared to junior residents. No interaction was found between training levels and level of sleepiness.

## DISCUSSION

We explored the relationships between subjective sleepiness, level of training, and overall sleep hygiene on adverse driving events after completion of an overnight shift in EM residents. The results show that high levels of subjective sleepiness were significantly associated with increased self-reported adverse driving events. Further, there was a significant increase in the level of sleepiness reported after completing the drive home compared to the level of sleepiness prior to driving home. Senior residents reported a lower number of adverse driving events compared to junior residents.

Our findings highlight the dangers of drowsy driving in an understudied and at-risk group, EM residents. Physicians in training are crucial to patient care at hospitals across the country. Interventions that improve sleep deprivation have been shown to reduce patient care errors made by these physicians.^16^ However, recognizing the effect of sleep deprivation on the safety and health of the physicians themselves needs to be brought to the forefront of our attention.

Research has shown that drowsy driving-related MVCs were more likely to involve individuals who average fewer hours of sleep per night, work overnight shifts, or have unusual work schedules.^17^ Working rotating shifts has been shown to result in higher levels of sleepiness compared to working overnight shifts exclusively.^18^ Based on these demographics, EM residents are inherently at high risk for sleep-related adverse driving events. The findings of our study support this hypothesis as 82% of participants reported experiencing an adverse driving event. This is concerning, and the results point to a very tangible injury risk for drowsy driving-related MVCs in this unique and vulnerable population.

Our results show that after working an overnight shift, EM residents reported significantly increased sleepiness after completing their drive compared to their sleepiness prior to initiating their drive home. The implications of this finding are of paramount importance. Immediately after completing an overnight shift, a resident may not recognize his or her true level of sleepiness and may feel safe to drive home. The increased degree of sleepiness after completion of the drive likely represents the true level of sleepiness. This suggests that emergency physicians in training may underestimate their degree of sleepiness immediately prior to initiating their drive home, putting them at risk for drowsy driving-related MVCs.

Our results also suggest that the subjective burden of sleep deprivation increases after performing a focused task (i.e., driving) particularly when coupled with working an overnight shift. A prior study conducted in the state of New York points to individual demographic characteristics as well as sleep, work, and driving patterns as key contributors to increasing drowsy driving. 19 Moreover, other studies show that adverse driving events occur after a longer duration of driving.^20^ Awareness of these facts is crucial. Understanding that the driver’s level of sleepiness upon completion of an overnight shift will increase throughout the duration of the drive is necessary in order to take meaningful corrective and preventive action to avoid drowsy driving.

Further, our results confirm that increased sleepiness is associated with a significantly greater number of adverse driving events. Past research shows that the best predictor of near-crash events is a prior episode of severe sleepiness at the wheel.^21^ Drowsy driving-related near-crash events are a strong predictive risk factor for future crashes.^22^ On average, the participants in our study reported that they felt “sleepy” at the end of their drive home. This alone puts these physician trainees at needless risk for future drowsy driving-related crashes.

These findings have important implications for residency training programs of all specialties and suggest that a multifaceted approach may be needed to address the problem and potential dangers of drowsy driving. First, recognition of this issue is the pivotal step toward enacting change. Our results show that junior residents reported more adverse driving events. It’s unlikely that new physicians have faced the regularity of drowsy driving at prior stages in their training. Upon entering residency, first-year residents should be intentionally educated about the dangers of drowsy driving. Second, residency training programs may need to adapt their culture to consider safer alternatives to drowsy driving for physician trainees of all levels.

Encouraging car pooling, creating call rooms for sleeping after an overnight shift, or identifying alternate methods of transportation home for residents are all possible solutions. Considering a consistent night-float system, as opposed to sporadic overnight shifts, is another possible avenue to explore. In this system, residents would work overnight shifts for an entire month at a time, allowing them to have more consistency in their sleep schedule. This would eliminate sporadic overnight shifts in other months. Resident physicians should be educated about their risk for drowsy driving and that their assessment of their level of sleepiness at the start of their drive may dangerously misrepresent their sleepiness at the end of their drive. Initiating a dialogue to promote wellness in this area is the feasible and viable first step to prevention. Graduate medical education departments across the country need to mandate better training for their residency programs in this area. To take the best care of patients throughout their careers, we must instill good habits in physicians at an early stage and implore residents to first take good care of themselves.

## LIMITATIONS

We realize that our study has a number of limitations. First, the participants were derived from a single training program. Although we were able to obtain a favorable distribution of residents across different training levels, the overall sample size was restricted by the total number of EM trainees within our program. Second, our study design relied on self-reported data and this approach could have inherently introduced social desirability and recall bias. As a result, some participants may have been both hesitant to report adverse driving events and may have incorrectly remembered whether they experienced an adverse driving event or not.

Third, it would be reasonable to consider that the recall bias could have been accentuated as a result of potentially greater drowsiness at the end of the drive. In turn, this might also affect the extent of social desirability bias if a participant realized he or she had a high (ie, more than expected) number of reported adverse driving events and chose to minimize some of their reporting. Nevertheless, prior research has shown that the subjective Karolinska Sleepiness Scale is positively correlated with objective measures of drowsiness.^23^ Future studies should consider building off our findings to obtain more objective data using high-fidelity driving simulation or a naturalistic driving research approach with a larger number of participants from several training programs.

## CONCLUSION

In EM residents driving home after completing an overnight shift, higher levels of subjective sleepiness were significantly associated with increased self-reported adverse driving events. Increased levels of sleepiness were reported after completing the drive home compared to the level of sleepiness reported prior to initiating the drive. Senior residents reported a lower number of adverse driving events compared to junior residents. Our findings emphasize the need to explore this relationship further to determine whether improvement in sleep hygiene or improved tolerance of sleepiness leads to fewer reported adverse events. Overall, these findings have important implications for the health and safety of physicians in training as well as the overall safety of the public.

## Supplementary Information







## Figures and Tables

**Table 1:** Proportion and distribution of self-reported adverse driving events with baseline characteristics of emergency medicine residents.

	Adverse driving				Adverse driving events$	

	Overall, n	Yes, n (%)	No, n (%)	P-value	Mean±SD	P-value
Levels of residency training						
Junior (PGY 1 + PGY 2)	24	21(88)	3(12)	0.64^a^	2.79±1.89	0.05^c^
Senior – PGY 3	14	11(79)	3(21)		2.57±1.83	
Senior – PGY 4	12	9(75)	3(25)		1.33±0.98	
Gender						
Male	32	28(88)	4(12)	0.25^a^	2.31±1.64	0.72^b^
Female	18	13(72)	5(28)		2.50±2.04	

Continuous independent variables	Overall, n	Yes, n (mean±SD)	No, n (mean±SD)	P-value	Coefficient, ß	P-value

Subjective sleepiness	50	41(13.37±2.35)	9(9.22±1.99)	0.00^b^	0.31	0.00^d^
Sleep hygiene	49	40(34.33±5.59)	9(34.11±5.11)	0.92^b^	0.06	0.20^d^

$ Adverse driving events were calculated by summing up each binary response (yes vs. no) from adverse driving event questions;

a Chi-square test (Note: the Fisher’s exact test was performed when 50% of the cells had counts less than 5);

b T test;

c Analysis of variance test;

d bivariate linear regression.

*SD*, standard deviation.

**Table 2:** Bivariate linear regression analyzing the relationship between sleepiness, sleep hygiene, and adverse driving events.

	Beta	P-value
Sleepiness - Sleep hygiene	0.06	0.40
Adverse driving events - Sleep hygiene	0.06	0.20
Adverse driving events - Sleepiness	0.31	<0.001

**Table 3:** Average level of sleepiness before and after driving home following an overnight shift.

	Sleepiness			

	Before (mean±SD)	After (mean±SD)	Difference$ mean (95%CI)	P-value#
Levels of subjective sleepiness	5.58±1.81	7.04±1.41	−1.46 (−1.93, −0.99)	<0.001

$ Difference = Average of level of sleepiness before minus average of level of sleepiness after driving home;

# A paired t test was used to compare the levels of subjective sleepiness before and after driving home.

*SD*, standard deviation; *CI*, confidence interval.

**Table 4:** Linear regression of adverse driving events on sleepiness adjusted for levels of residency training.

	Adverse driving events	

	Beta	P-value
Levels of subjective sleepiness	0.30	<.001
Levels of residency training	−0.59	0.003
