# Supplementary material for: The Association of Sleep Hygiene and Drowsiness with Adverse Driving Events in Emergency Medicine Residents
Source: West J Emerg Med. 2020 Oct 27;21(6):219–24. doi: 10.5811/westjem.2020.8.47357 (PMC7673877; doi:10.5811/westjem.2020.8.47357)
Supplement: Supplementary file 1 [file wjem-21-219-s001.docx]

**Appendix 1**

**Karolinska Sleepiness Scale (KSS).**

| Extremely alert | 1 |
| --- | --- |
| Very alert | 2 |
| Alert | 3 |
| Rather alert | 4 |
| Neither alert or sleepy | 5 |
| Some signs of sleepiness | 6 |
| Sleepy, but no effort to keep awake | 7 |
| Sleepy, but some effort to keep awake | 8 |
| Very sleepy, great effort to keep awake, fighting sleep | 9 |
| Extremely sleepy, can’t keep awake | 10 |

A ̊kerstedt T, Gillberg M. Subjective and objective sleepiness in the active individual. Int J Neurosci 1990;52:29–37

**Karolinska Sleepiness Scale:** Applied to question of sleepiness ***prior*** to driving home.

| To the best of your ability, please rate your level of sleepiness immediately after your overnight shift **PRIOR TO YOUR DRIVE HOME** | |
| --- | --- |
| Extremely alert | 1 |
| Very alert | 2 |
| Alert | 3 |
| Rather alert | 4 |
| Neither alert or sleepy | 5 |
| Some signs of sleepiness | 6 |
| Sleepy, but no effort to keep awake | 7 |
| Sleepy, but some effort to keep awake | 8 |
| Very sleepy, great effort to keep awake, fighting sleep | 9 |
| Extremely sleepy, can’t keep awake | 10 |

**Karolinska Sleepiness Scale:** Applied to question of sleepiness ***after*** driving home.

| To the best of your ability, please rate your level of sleepiness **AFTER YOU COMPLETED YOUR DRIVE HOME** | |
| --- | --- |
| Extremely alert | 1 |
| Very alert | 2 |
| Alert | 3 |
| Rather alert | 4 |
| Neither alert or sleepy | 5 |
| Some signs of sleepiness | 6 |
| Sleepy, but no effort to keep awake | 7 |
| Sleepy, but some effort to keep awake | 8 |
| Very sleepy, great effort to keep awake, fighting sleep | 9 |
| Extremely sleepy, can’t keep awake | 10 |

We calculated subjective sleepiness of residents before and after driving home adapted to the KSS. We summed up the scores obtained from PRIOR TO DRIVE HOME and AFTER YOU DRIVE HOME as total subjective sleepiness. Higher scores indicate higher levels of sleepiness.
