# Supplementary material for: The Association of Sleep Hygiene and Drowsiness with Adverse Driving Events in Emergency Medicine Residents
Source: West J Emerg Med. 2020 Oct 27;21(6):219–24. doi: 10.5811/westjem.2020.8.47357 (PMC7673877; doi:10.5811/westjem.2020.8.47357)
Supplement: Supplementary file 2 [file wjem-21-219-s002.docx]

**Appendix 2**

**Adverse Driving Events Questionnaire**

| During your drive home, did any of the following occur while you were driving? | | | |
| --- | --- | --- | --- |
|  | No | Yes | I did not drive home |
| Used a technique to keep yourself more awake (loud music, rolled down windows, made a phone call, etc) |  |  |  |
| Drifted out of your lane |  |  |  |
| Felt your car go over the rumble strips |  |  |  |
| Found yourself following too closely to the car in front of you |  |  |  |
| Unexpectedly pressed on your brakes harder than usual |  |  |  |
| Had to break hard to avoid rear ending a vehicle in front of you |  |  |  |
| Went through an intersection when the light was turning red |  |  |  |
| Ran a stop sign |  |  |  |
| Felt your eyes get heavy |  |  |  |
| Rested your eyes |  |  |  |
| Pulled over to take a nap |  |  |  |
| Dozed off or fell asleep while the car was stopped |  |  |  |
| Dozed off or fell asleep while driving |  |  |  |
| Had a near car accident (narrowly avoided crash) |  |  |  |
| Had a car accident |  |  |  |

The number of adverse driving events were calculated by summing up each binary response (YES=1 vs. NO=0).
