# Supplementary material for: The Association of Sleep Hygiene and Drowsiness with Adverse Driving Events in Emergency Medicine Residents
Source: West J Emerg Med. 2020 Oct 27;21(6):219–24. doi: 10.5811/westjem.2020.8.47357 (PMC7673877; doi:10.5811/westjem.2020.8.47357)
Supplement: Supplementary file 3 [file wjem-21-219-s003.docx]

**Appendix 3**

**Sleep Hygiene Index**

| Please rate the following statements regarding your usual sleep habits | | | | | |
| --- | --- | --- | --- | --- | --- |
|  | Never (1 pts) | Rarely (2 pts) | Sometimes (3 pts) | Frequently (4 pts) | Always (5 pts) |
| I take daytime naps lasting two or more hours |  |  |  |  |  |
| I go to bed at different times from day to day |  |  |  |  |  |
| I get out of bed different times from day to day |  |  |  |  |  |
| I exercise to the point of sweating within one hour prior to bedtime |  |  |  |  |  |
| I stay in bed longer than I should two to three times per week |  |  |  |  |  |
| I use alcohol, tobacco, or caffeine within four hours of going to bed or after going bed |  |  |  |  |  |
| I do something that may wake me up before bedtime (for example: play video, games, use the internet, or clean) |  |  |  |  |  |
| I go to bed feeling stressed, angry, upset, or nervous |  |  |  |  |  |
| I use my bed for things other than sleeping or sex (for example: watch television, read, eat, or study) |  |  |  |  |  |
| I sleep on an uncomfortable bed (for example: poor mattress or pillow, too much or not enough blankets) |  |  |  |  |  |
| I sleep in an uncomfortable bedroom (for example: too bright, too stuffy, too hot, too cold, or too noisy) |  |  |  |  |  |
| I do important work before bedtime (for example: pay bills, schedule, or study) |  |  |  |  |  |
| I think, plan, or worry when I am in bed |  |  |  |  |  |

These 13 items used to assess sleep hygiene status were originally derived from the diagnostic criteria for inadequate sleep hygiene in the International Classification of Sleep Disorders. The score of each item was summed up to deliver a holistic assessment of sleep hygiene habits. Higher total scores indicate a worse degree of sleep hygiene status

Mastin DF, Bryson J, Corwyn R. Assessment of sleep hygiene using the Sleep Hygiene Index. J Behav Med.

2006;29(3):223-7.
